# Gremlin2 Activates Fibroblasts to Promote Pulmonary Fibrosis Through the Bone Morphogenic Protein Pathway

**DOI:** 10.3389/fmolb.2021.683267

**Published:** 2021-06-28

**Authors:** Caijuan Huan, Wangting Xu, Yaru Liu, Kexin Ruan, Yueli Shi, Hongqiang Cheng, Xue Zhang, Yuehai Ke, Jianying Zhou

**Affiliations:** ^1^ Department of Respiratory Medicine, The First Affiliated Hospital, Zhejiang University School of Medicine, Hangzhou, China; ^2^ Department of Pathology and Pathophysiology, Zhejiang University School of Medicine, Hangzhou, China; ^3^ The Fourth Affiliated Hospital, Zhejiang University School of Medicine, Yiwu, China

**Keywords:** IPF (idiopathic pulmonary fibrosis), gremlin2, BMP antagonists, P-smad1, fibroblast

## Abstract

Idiopathic pulmonary fibrosis (IPF) is a progressive lung disease causing unremitting extracellular matrix deposition. Transforming growth factor-β (TGF-β) superfamily involves bone morphogenetic proteins (BMPs) and TGF-β, and the balance between the activation of TGF-β-dependent SMADs (Smad2/3) and BMP-dependent SMADs (Smad1/5/8) is essential for fibrosis process. *GREM2*, initially identified as a TGF-β-inducible gene, encodes a small secreted glycoprotein belonging to a group of matricellular proteins, its role in lung fibrosis is not clear. Here, we identified Gremlin2 as a key regulator of fibroblast activation. Gremlin2 was highly expressed in the serum and lung tissues in IPF patients. Bleomycin-induced lung fibrosis model exhibited high expression of Gremlin2 in the bronchoalveolar lavage fluid (BALF) and lung tissue. Isolation of primary cells from bleomycin-induced fibrosis lung showed a good correlation of Gremlin2 and Acta2 (α-SMA) expressions. Overexpression of Gremlin2 in human fetal lung fibroblast 1 (HFL-1) cells increased its invasion and migration. Furthermore, Gremlin2 regulates fibrosis functions through mediating TGF-β/BMP signaling, in which Gremlin2 may activate TGF-β signaling and inhibit BMP signaling. Therefore, we provided *in vivo* and *in vitro* evidence to demonstrate that Gremlin2 may be a potential therapeutic target for the treatment of IPF.

## Introduction

Idiopathic pulmonary fibrosis (IPF), is the most common form of interstitial pneumonia (ATS/ERS, 2000), which deteriorates rapidly in a short period of time (Martinez et al., 2005) and the median survival is only 2–3 years (Raghu et al., 2011).Tissue fibrosis is an increasing cause of severe morbidity and mortality with limited therapeutic options, and IPF is characterized by patchy subpleural parenchymal fibrosis with pathological features including the accumulation of myofibroblasts, the formation of fibroblast foci, distortion of pulmonary architecture, and increased collagen deposition (Noble et al., 2012). Mechanisms leading to severe and progressive fibrosis are not entirely understood. Previous studies demonstrated that fibroblasts from patients with IPF acquire an invasive phenotype that is essential for severe fibrogenesis (Li et al., 2011; Lovgren et al., 2011; Ahluwalia et al., 2016; Chen et al., 2016).

Fibroblasts are the main effector cells in fibrosis. They migrate from different sources to damaged sites, such as resident stromal fibroblasts, circulating fibroblasts, as well as epithelial cells and pericytes, and they are eventually activated as myofibroblasts (Andersson-Sjöland et al., 2008; Fernandez and Eickelberg, 2012; Wolters et al., 2014; Bagnato and Harari, 2015), which secrete excessive extracellular matrix (ECM), resulting in increased tissue hardness and loss of alveolar tissue function.

The progression of fibrosis is regulated by the transforming growth factor-β (TGF-β)/bone morphogenic protein (BMP) family.

After an injury to the airway epithelium, important profibrotic mediators, such as TGF-β, are released from many types of lung cells, involving myofibroblasts and epithelial cells (Li et al., 2017). Their regulation on progenitor cells also affects fibrosis progression. TGF-β is very important for the progression of pulmonary fibrosis in mice, as it regulates fibroblast proliferation, collagen synthesis and myofibroblast differentiation (Bonniaud et al., 2004; Bonniaud et al., 2005; Kang et al., 2007).The other important fibrosis regulator is BMP family, especially BMP4/7, which is considered to be an important regulatory molecule for anti-fibrosis (Hinck and Huang, 2013; Huan et al., 2015; Chanda et al., 2019). In recent years, antagonists of the BMP family gradually become the center of research for fibrosis treatment. For example, Gremlin1 and Follistatin-like 1 were proved to participate in and affect pulmonary fibrosis (Dong et al., 2015; Church et al., 2017; Myllärniemi, et al., 2008a; Mezzano et al., 2018).

Overall, the balance between the activation of BMP-dependent SMADs (SMAD1/5/8) and TGF-β-dependent (SMAD2/3) plays a great role in fibrosis process.

Gremlin2 (*GREM2*), also known as PRDC, located on human chromosome 1Q43 (CKTSF1B2) (Katoh and Katoh, 2004), is a highly conserved secretory glycoprotein in the DAN region. Gremlin2 was first discovered as a protein associated with embryonic development (Hsu et al., 1998; Stanley et al., 1998). In recent years, its highly stable dimer structure formed via non-disulfide bonds has led scientists to further explore its structure (Kattamuri et al., 2012; Hinck and Huang, 2013; Nolan et al., 2016). Heparin-competitive antagonistic combination of Gremlin2 and BMP provides a new idea for Gremlin2 targeted therapy and the exploration of its structure (Nolan et al., 2013; Kattamuri et al., 2017). As a development-related protein, Gremlin2 is highly expressed during embryonic development but its high levels in adults may imply a severe disease state. It is known that Gremlin2 plays a crucial role in kidney development and kidney injury (Wen et al., 2019). Furthermore, as a strong antagonist of the BMP family, Gremlin2 has a strong effect on gastric cancer and myocardial fibrosis, but its effect on pulmonary fibrosis has not been reported (Sanders et al., 2016; Ran et al., 2019). However, the ability of Gremlin2 to regulate fibroblast activity in pathological settings has not been investigated.

Our previous study showed that Gremlin2 is highly expressed in myofibroblasts in single-cell sequencing (Xie et al., 2018). Data showed Gremlin2 expression is significantly high in myofibroblasts, which is consistent with specific myofibroblast markers Acta2 and Myh11 (Hsia et al., 2016; Hinz et al., 2007). Meanwhile, in other mesenchymal cells, Gremlin2 expression is low, indicating that Gremlin2 has a functional role in myofibroblasts.

Therefore, here, we aimed to show the effect of Gremlin2 in IPF via *in vivo* and *in vitro* studies, as well as through database investigations and understand the potential of it as a therapeutic target.

## Materials and Methods

### Mice

C57/BL6 male mice (6–8 weeks old) were purchased from GemPharmatech Co., Ltd., Jiangsu, China. All mice were housed and cared for in a pathogen-free facility at Zhejiang University. All animal protocols were approved by the animal care and use committee of the Zhejiang University School of Medicine.

Lungs were removed from mice at the age of 8-12 weeks after appropriate treatment. The tissues were minced, digested, and cultured in DMEM supplemented with 10% fetal bovine serum (FBS) and antibiotics-antimycotics (Thermo Fisher Scientific).

### Idiopathic Pulmonary Fibrosis Samples From Patients

Lung tissue samples were obtained from surgical lung biopsies or lung transplant explants from patients with IPF or other types of pulmonary fibrosis. IPF diagnosis was performed according to standard accepted American Thoracic Society recommendations (ATS/ERS, 2000). Human blood samples were obtained from the Clinical Laboratory. All samples were collected from the First Affiliated Hospital of Zhejiang University.

### Bleomycin Administration and Bronchoalveolar Lavage Fluid

Bleomycin was injected intratracheally at 2.5 U/kg body weight. Mice exposed to the same volume of PBS were used as controls and sacrificed via pentobarbital injection after 7, 14, and 21 days. Lungs were harvested for RNA preparation, protein isolation or fibroblast isolation. For bronchoalveolar lavage, the trachea was lavaged three times with 0.8 ml sterile saline at room temperature. Samples were centrifuged at 1,500 rpm for 5 min, and the supernatant was collected and stored at −80°C until further use.

### Enzyme-Linked Immunosorbent Assay of Serum and Bronchoalveolar Lavage Fluid

The mice were anesthetized with 1% pentobarbital at a dose of 50 mg/kg, and the apical blood was obtained in a sterile environment and kept at 4°C overnight. The next day, the blood was centrifuged at 4°C for 15 min at 3,000 × g. The supernatant was removed and stored separately below −70°C before detection. BALF from each mouse was collected as mentioned in 2.3. Before measurement, the BALF supernatant of each mouse was stored at −70°C. Gremlin2 levels in the serum and BALF supernatant were measured using Mouse Gremlin-2 ELISA Kit (Biolebo, Beijing, China), according to the manufacturer's instructions.

### Lung Histology and Immunohistochemistry

Mice were sacrificed at different time points after bleomycin treatment under anesthesia. The left lung of each mouse was fixed with 10% neutral buffer formalin overnight, then embedded in paraffin using standard procedures. Tissues were sectioned to 5 μm slices for hematoxylin and eosin, Masson’s Trichrome, and Gremlin2 staining (Table 1).

**TABLE 1:** Reagent and antibody.

Product name	Company	Catalog	Lot
Rabbit polyclonal to PRDC-C-terminal	Abcam, cambridge, MA	Cat#ab228736	Lot# GR3209267-6
GREM2 antibody	CUSABIO	Catalog#CSB-PA107590	Lot#10902Y
Rabbit polyclonal to fibronectin	Abcam, cambridge, MA	Cat# ab2413, RRID:AB_2262874	Lot#GR3323518-2
Anti-alpha smooth muscle actin antibody	Abcam, cambridge, MA	Cat# ab7817, RRID:AB_262054	Lot# GR3257713-10
Mouse PRDC/GREM2 antibody	R And D systems	Cat# AF 2069, RRID:AB_2263614	Lot#USW0114091
Mouse PRDC/GREM2 biotinylated antibody	R And D systems	Cat# BAF 2069, RRID:AB_2279268	Lot#UUB0112051
Phospho-Smad1(Ser463/465)	Cell signaling TECHNOLOGY	Cat# 13820T	Lot#3
/Smad5(Ser463/465)			
/Smad9(Ser465/467)			
(D5B10)Rabbit mAb			
Smad1(D59D7)Rabbit mAb	Cell signaling TECHNOLOGY	Cat# 6944T	Lot#5
Phospho-SMAD2(Ser465/467) (E8F3R)	Cell signaling TECHNOLOGY	Cat# 188338T	Lot#3
Smad2 (D43B4) XP^®^ rabbit mAb	Cell signaling TECHNOLOGY	Cat#5339T	Lot#6
Rabbit anti-P-Smad3(S423/425)		Cat# ET1609	Lot#HM0708
Human Gremlin-2 ELISA kit	Biolebo, beijing, China	ZN2207	Lot#TF0611
Recombinant human PRDC/GREM2 protein, CF	R&D systems	Catalog #8436-PR-050	Lot#DEUB0220071
HRP labeled goat anti-rabbit IgG(H + L)	BIOKER BIOTECHNOLOGY	Catalog #BK-R050	Lot#2313

The alveolar wall thickness and alveolar space were photographed and measured using DP2-BSW software (Olympus, Tokyo, Japan). Slides were screened in a blinded manner and six lungs were observed in areas that did not contain bronchi or major pulmonary vessels. A total of 20 random measurements of alveolar wall thickness were recorded in each field.

### Immunohistochemistry Score

We selected five sections randomly from each slide at 200× magnification for IHC score statistics as follows: negative, 1 point (less than 5% of cells); weak positive 2 points (5–25% of cells); positive, 3 points (25–50% of cells); strong positive, 4 points (50–75% of cells). IHC score staining degree and multiple cell ratio score of each section were added, the average of the five visual fields was accepted as the IHC score of the slide, and the average score of each slide was taken after two investigators agreed.

### Immunofluorescence

For cell immunofluorescence, after 48 h of TGF-β1 stimulation, fibroblasts were washed in PBS preheated at 37°C, fixed in 4% paraformaldehyde for 20 min, and permeated with 0.1% Triton X-100 for 20 min. Cells were blocked in 10% FBS at room temperature for 1 h, and fibroblasts were incubated overnight with antibodies against Gremlin2 or α-SMA (Table 1). On the second day, fibroblasts were washed with PBS, and 488 goat anti-rabbit/rat IgG (Invitrogen, Fisher Scientific, California, United States) was added for 1 h at room temperature. Cells were then washed with PBS, then immersed in DAPI to stain the nuclei. The staining was performed using a positive two-photon confocal microscope (Olympus BX61).

For tissue immunofluorescence, paraffin sections were baked, dewaxed, and hydrated after microwave antigen repair. They were then incubated in 0.5% Triton-TBS for 20 min and 5% goat serum for 30 min. Anti-Gremlin2 and α-SMA antibodies were added at 4°C overnight. The next day, slides were washed with PBS and incubated for 2 h at room temperature with 488/576 goat anti-rabbit/rat IgG (Invitrogen, Fisher Scientific, California, United States), then washed with PBS, and immersed in DAPI to observe the nuclei. Imaging was performed using a positive two-photon confocal microscope (Olympus BX61).

### Quantitative Real-Time Polymerase Chain Reaction

Total RNA was extracted from tissues or cells using TRIzol (Invitrogen, Carlsbad, CA, United States) according to the manufacturer’s protocol. RNA concentration was measured using a spectrophotometer (Eppendorf, Hamburg, Germany). Reverse transcription of total RNA (2 g) 20 μL was performed using PrimeScript II 1st Strand cDNA Synthesis Kit (Takara, Otsu, Japan). Quantitative real-time PCR was performed using FastStart universal SYBR Green Master Kit (Mannheim Roche, Germany). Sequences of specific primers used are shown in Table 2.

**TABLE 2:** Sequences of specific primers.

	Sense strand (5′ to 3′)	Anti-sense strand (5′ to 3′)
Gremlin2(mouse)	GGT​AGC​TGA​AAC​ACG​GAA​GAA	TCT​TGC​ACC​AGT​CAC​TCT​TGA
GAPDH(mouse)	AAT​GGA​TTT​GGA​CGC​ATT​GGT	TTT​GCA​CTG​GTA​CGT​GTT​GAT
Col1a1(mouse)	CCA​AGA​AGA​CAT​CCC​TGA​AGT​CA	TGCACGTCATCGCACACA
Col1a2(mouse)	CGGAGAAGCTGGATCTGC	CAG​GAG​GAC​CCA​TTA​CAC​CA
Gremlin2(human)	ATC​CCC​TCG​CCT​TAC​AAG​GA	TCT​TGC​ACC​AGT​CAC​TCT​TGA
GAPDH(human)	TGT​GGG​CAT​CAA​TGG​ATT​TGG	ACA​CCA​TGT​ATT​CCG​GGT​CAA​T
Col1(human)	GTT​GCT​GCT​TGC​AGT​AAC​CTT	AGG​GCC​AAG​TCC​AAC​TCC​TT
ACTA2(human)	GAC​AAT​GGC​TCT​GGG​CTC​TGT​AA	CTG​TGC​TTC​GTC​ACC​CAC​GTA
Fibronectin(human)	CAG​GAT​CAC​TTA​CGG​AGA​AAC​AG	GCC​AGT​GAC​AGC​ATA​CAC​AGT​G

### Western Blotting

The cells were washed with cold PBS and lysed with NP−40, then 6X loading buffer was added. Equivalent proteins were separated via SDS-PAGE, then transferred to a cellulose nitrate membrane (Pall, Port Washington, NY) and incubated with the relevant primary antibodies (anti-Gremlin2, anti-α-Tubulin, anti-fibronectin, anti-collagen1, anti-α-SMA). This protocol was performed in three replicates. Shorter exposures were selected for optical density analysis to ensure that the band strength was within the linear range, and the integral density of the specified band was calculated using the ImageJ software.

### Transwell Assay

For cell migration, 5 × 10^4^ cells were resuspended in 200 μl starvation medium and seeded into the upper chamber. In the lower chamber, 600 μl of complete medium containing 10% FBS was added. After 24 h, the medium was removed, the upper chamber was washed twice with PBS, and cells were fixed with 4% PFA at room temperature for 30 min. The cells were stained with crystal violet. Three fields were randomly selected under 100× magnification and cells were counted in each field for statistical analysis.

For cell invasion, the matrigel was diluted in Ham’s F-12K (Kaighn’s) Medium (1:32) (Gibco, Thermo Fisher, United States), added to the upper chamber one day in advance, and blow-dried overnight under ultraviolet light. 2 × 10^5^ cells were resuspended in 100 μl starvation medium and added to the superior lumen. Exactly 600 μl of medium containing 10% FBS was added to the lower chamber. After 48 h, the cells were collected, and the same steps were performed as the migration experiment.

### GEO Database Analysis

The original data in the GEO database were homogenized. We used R language (R i386 3.6.1.Ink) and GEO2R analysis software provided by the GEO website for group analysis. Then, the data for different groups were plotted using GraphPad Prism (version 5) and paired t-test was performed for statistical analysis.

### Lentiviral Infection

To knock out *GREM2* in fibroblasts, shRNA-Gremlin2-plko.1 and a control lentiviral vector [(RRID:Addgene_23,260), Sigma-Aldrich] were obtained. 293T (RRID:CVCL_LF41) cells were transfected with pMD2. G and psPAX2 were prepared using Lipofectamine 3,000 reagent (Invitrogen, Addgene, Cambridge, MA). After 48 and 72 h, the virus superfluids were collected and filtered through a 0.22 μm filter, and added to fibroblasts. After 12 h, the culture medium was changed to allow the cells to grow until treatment and analysis.

### Statistical Analysis

All the results were expressed as the mean ± SD, calculated using the statistical program of GraphPad Prism version 5. Student’s t-test (two-tailed) was used and *p* < 0.05 was considered statistically significant.

## Results

### High Expression of Gremlin2 has a Strong Correlation With Idiopathic Pulmonary Fibrosis in the Fibrotic Area

**FIGURE 1:**
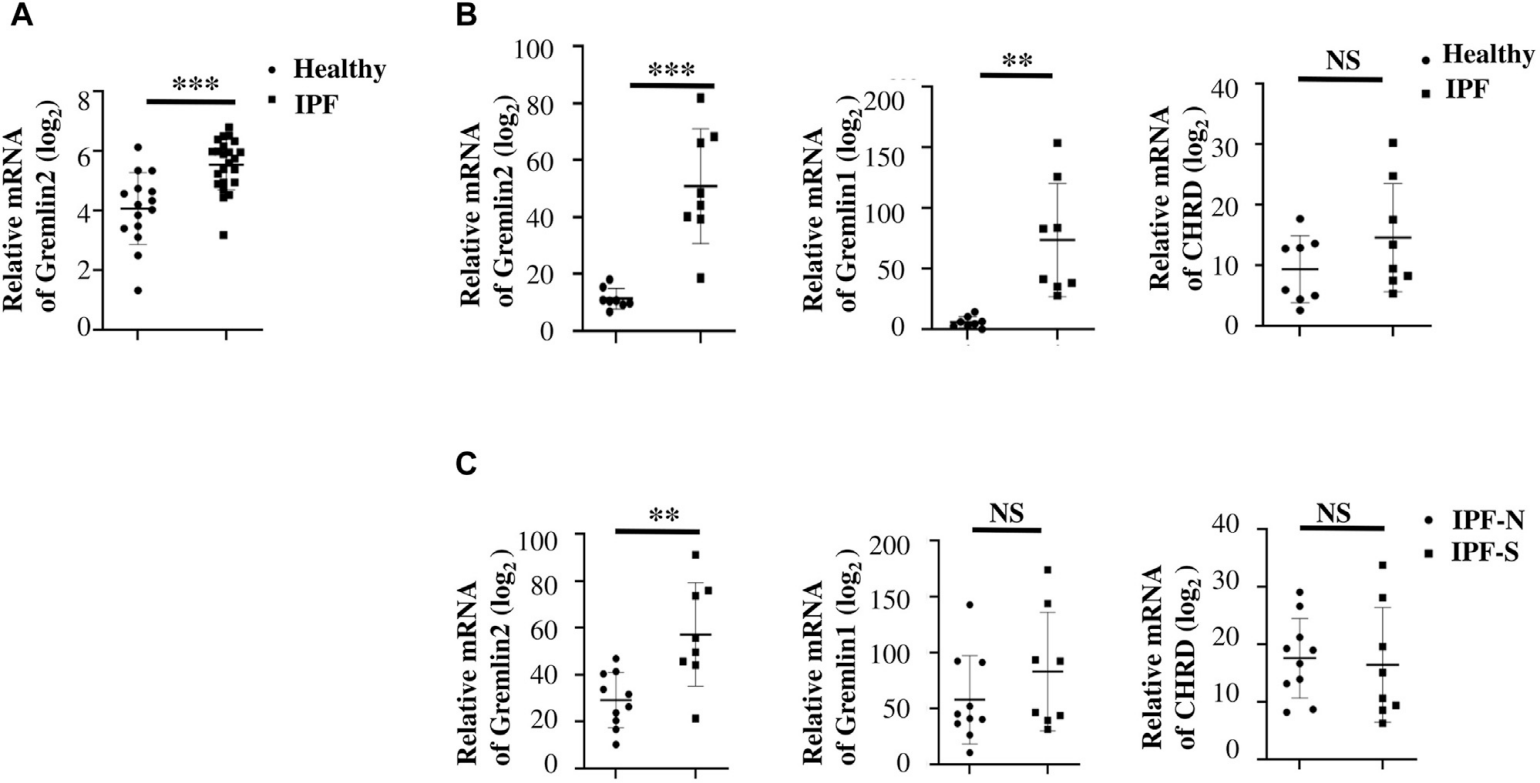
High expression of Gremlin2 in the fibrotic area obtained from IPF databases **(A)** The mRNA expression of Gremlin2 in patients with IPF and healthy people in database GSE10667. The data has been log_2_ processed. **(B)** The mRNA expression levels of Gremlin2, Gremlin1, and CHORD in the lung tissues of eight IPF patients and eight healthy people using the GSE99621 database. The data has been log_2_ processed. **(C)** mRNA expression levels of Gremlin2, Gremlin1, and CHORD in the GSE99621 database in the scar regions of eight IPF patients and ten non-scar regions of patients with IPF. The data has been log2 processed.NS stands for *p* > 0.05, *p < 0.05, **p < 0.01, ***p < 0.001. Student’s t-test was used for statistics. IPF-N, non-fibrosis lung area of IPF patients; IPF-S, fibrosis lung area of patients with IPF.

To discern the importance of Gremlin2 in pulmonary fibrosis, we evaluated the expression of Gremlin2 using the GEO database and selected two fibrosis-related databases, GSE99621 (https://www.ncbi.nlm.nih.gov/geo/query/acc.cgi?acc=GSE99621) and GSE10667 (https://www.ncbi.nlm.nih.gov/geo/query/acc.cgi). GSE10667 exhibited that the transcription level of Gremlin2 in the lung tissues of patients with IPF was significantly higher than that of healthy people (Figure 1A, *p* < 0.001). In GSE99621, we divided the data into Group1 (Figure 1B), patients with fibrosis, and healthy controls, and Group2 (Figure 1B), fibrosis foci and non-fibrosis foci of patients. Gremlin1 (*p* = 0.0011) (Myllärniemi et al., 2008a; Myllärniemi et al., 2008b) and Gremlin2 (*p* < 0.001) expressions in Group 1 (Figure 1B) were significantly high in the lung tissues of patients with fibrosis, while there was no difference in the expression of another BMP strong antagonist CHRD (*p* = 0.1819) between healthy people and patients with fibrosis. In Group2 (Figure 1C), only Gremlin2 (*p* = 0.032) expression was significantly higher in the fibrotic scar area than that in the non-fibrotic scar area of patients with IPF. Meanwhile, Gremlin1 (*p* = 0.2622) and CHRD (*p* = 0.7755) expressions showed no statistical significance. These two databases suggested that there was a strong relationship between Gremlin2 and IPF.

### Gremlin2 Expression is Increased in Patients With Idiopathic Pulmonary Fibrosis

**FIGURE 2:**
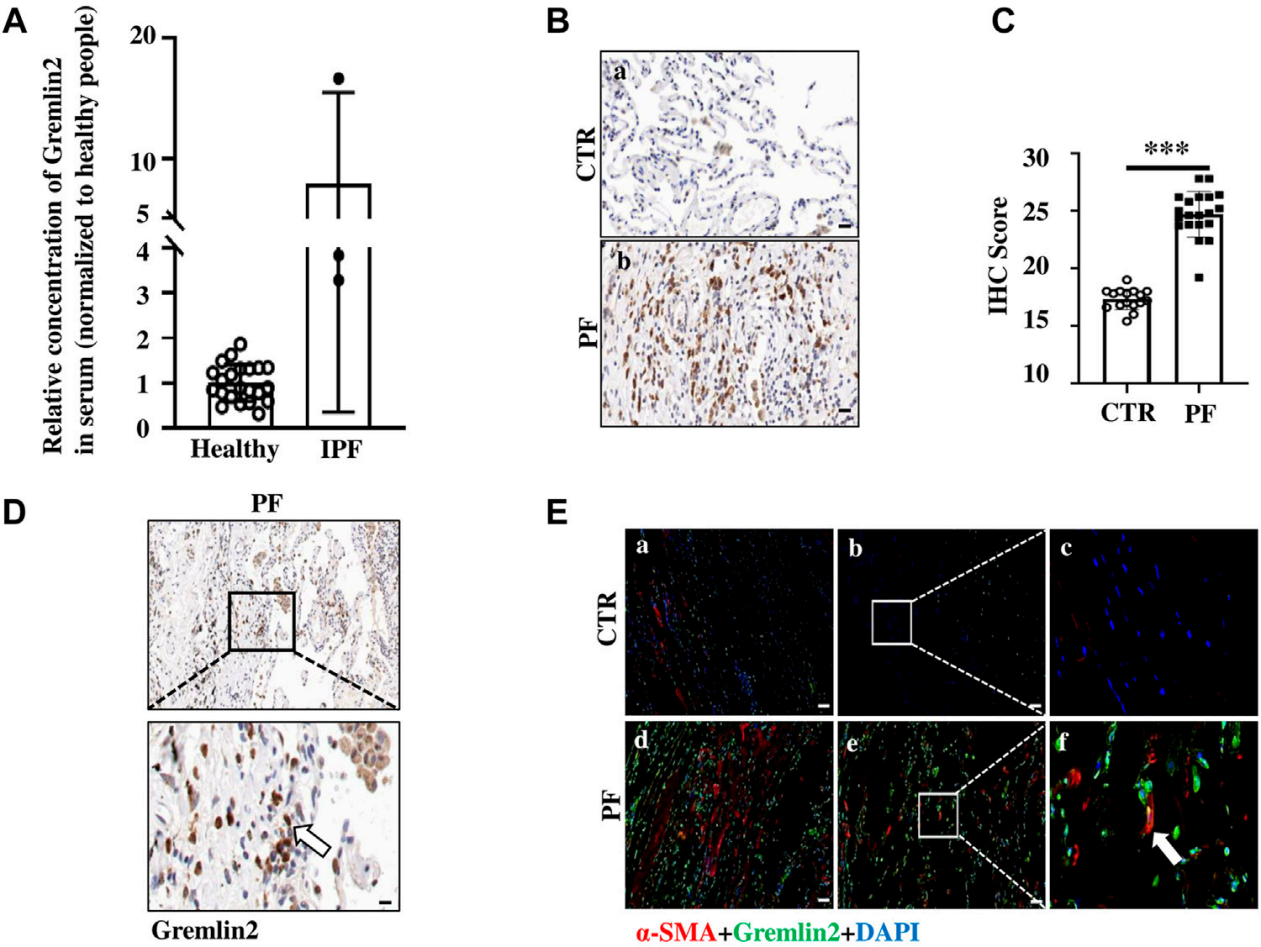
Gremlin2 expression is increased in patients with IPF **(A)** Expression of Gremlin2 in the blood serum detected using ELISA, CTR (healthy control) *n* = 22, IPF *n* = 3. **(B)** Representative pictures of IHC showing Gremlin2 expression in the lung tissue. Scale Bar = 50 μm. **(C)** IHC score of patients via immunohistochemical staining grading, CTR (normal) *n* = 16, PF *n* = 20, ***p < 0.001, paired t-test. **(D)** Representative photos of Gremlin2 immunohistochemical staining in the fibrotic lung tissue samples of the patient. Lower graph shows the strong positive area of Gremlin2 magnified from upper graph, white arrow shows the strong positive cells of Gremlin2. Scale Bar = 20 μm. **(E)** Gremlin2 was co-localized with α-SMA in fibrotic foci. Representative images of immunofluorescence detection for α-SMA (red) and gremlin2 (green), and merged (yellow) images of normal subjects and patients with IPF. Graph **(2Ea–c)** showed the pulmonary tissue in the fibrotic area of normal lung tissues near the fibrotic area of patients with IPF; graph **(2Ed,e)**, f showed the fibrotic lung tissue of patients with IPF; graph **(2Ec,f)** showed the locally enlarged view in the white box in graph **(2Eb,e)**; white arrow points to the cells with high co-expression of α-SMA and Gremlin2. CTR is the normal tissue around the pulmonary tissue of patients with fibrosis, and PF is the lung tissue section of patients with lung fibrosis. Scale Bar = 50 μm. NS stands for *p* > 0.05, *p < 0.05, **p < 0.01, ***p < 0.001. Student’s t-test was used for statistics.

To verify the data obtained from databases, we collected blood samples from four patients with non-IPF fibrosis and three patients with IPF, and measured the concentration of Gremlin2 in the blood serum. These IPF patients were first diagnosed with IPF and had not received anti-fibrosis therapy. The concentration of Gremlin2 in the peripheral blood serum was detected using ELISA (Figure 2A), which showed that Gremlin2 expression was high in patients with IPF and low in non-IPF patients.

To further detect the expression of Gremlin2 in the lung tissue, IHC was performed using the lung tissue samples from 20 patients with fibrosis, and the expression of Gremlin2 was compared with that of 16 healthy lung tissues. The patient information is shown in Table 3. Gremlin2 expression was hardly detected in healthy lung tissues but was high in the fibrotic foci (Figure 2B). IHC score (Figure 2C) showed that Gremlin2 expression was statistically high in fibrotic lung tissues (*p* < 0.001). To verify whether myofibroblasts are the main effector cells of Gremlin2, we detected the morphology of cells with high Gremlin2 expression (Figure 2D) and co-stained cells with a myofibroblasts marker. We observed the strong positive cells, which showed the long spindle shape characteristic of myofibroblast cells and were concentrated in the fibroblast lesions (Figure 2D). At the same time, Gremlin2 and α-SMA expressions were observed in the adjacent regions (Figure 2E). The precise localization of Gremlin2 is somewhat difficult, as it is a secreted protein, just like another secreted protein IL-11 (Ng et al., 2019).

**TABLE 3:** Patients’ information of IPF and other types of pulmonary fibrosis.

IHC of patients’ slices				
Pathology number	Gender	Age (years old)	Pathological diagnosis	Sample site
2019106687–5	Male	51	Pulmonary fibrosis nodules with charcoal deposition	Middle lobe of right lung
2019106687–6				Adjacent area of fibrosis
2019112471–3	Male	40	Fibrotic nodules	Inferior lobe of right lung
2019112471–5				Adjacent area of fibrosis
2019112971–3	Female	56	Chronic inflammatory fibrosis	Upper lobe of left lung
2019112971–4				Adjacent area of fibrosis
2019116608–9	Female	72	Fibrotic nodules	Inferior lobe of right lung
2019116608–8				Adjacent area of fibrosis
2019117672–9	Male	67	Chronic mucosal inflammation with pulmonary fibrosis nodules	Upper lobe of right lung
2019117672–13				Adjacent area of fibrosis
2020000937–3	Female	65	Fibrotic nodules	Middle lobe of right lung
2020000937–4				Adjacent area of fibrosis
2020001297–2	Female	49	Fibrotic nodules	Upper lobe of left lung
2020001297–5				Adjacent area of fibrosis
2020003176–2	Female	69	Fibrotic collagenous nodules	The proper segment of the upper lobe of the left lung
2020003176–3				Adjacent area of fibrosis
2020006458–10	Male	55	Chronic inflammation of lung tissue with collagen fibrotic nodules	Inferior lobe of left lung
2020006458–11				Adjacent area of fibrosis
2020009101–2	Male	44	Chronic inflammation of lung tissue with fibrotic nodules	Inferior lobe of right lung
2020009101–3				Adjacent area of fibrosis
2020009493–3	Male	51	Fibrotic nodules	Upper lobe of right lung
2020009493–8				Adjacent area of fibrosis
2020009765–3	Female	74	Chronic inflammatory fibrosis	Upper lobe of right lung
2020009765–7				Adjacent area of fibrosis
2020012831–1	Male	55	Fibrosis and degenerative necrotic nodules	Inferior lobe of right lung
2020012831–2				Adjacent area of fibrosis
2020020563–3	Male	70	Fibrotic nodules with charcoal deposition	Upper lobe of left lung
2020020563–4				Adjacent area of fibrosis
2020037421–1	Male	68	Interstitial pulmonary fibrosis	Inferior lobe of right lung
2020037421–2				Adjacent area of fibrosis
201651141	Male	64	Chronic interstitial pneumonia with fibrosis	Right lung
201709433	Female	69	Chronic interstitial pneumonia with fibrosis	Right lung
201713228	Male	67	Chronic interstitial pneumonia with fibrosis	Right lung
201722515	Male	58	Interstitial pulmonary fibrosis	Right lung

**Blood serum of IPF patients**				
**Medical record number**	**Gender**	**Age (years old)**	**Clinical diagnosis**	**Illness stage**
5057536	Female	62	IPF	Recurring fever with fatigue for a year, acute exacerbation
3165154	Female	66	IPF	Repeated cough for three years, chest tightness for two years, aggravation for half a year, with respiratory failure
4141982	Male	83	IPF	Repeated shortness of breath for three years, acute exacerbation with respiratory failure for one week

These clinical data were consistent with the databases analysis results, suggesting the possibility of clinical transformation of Gremlin2.

### Gremlin2 Expression is High in Bleomycin-Induced Mice Lung Tissues

We treated C57BL/6 mice with bleomycin (Tashiro et al., 2017) and detected the expression levels of Gremlin2 at mRNA and protein levels in the lung tissue. Since Gremlin2 is a secretory glycoprotein, we collected mouse alveolar lavage fluid for the detection of Gremlin2 concentration using ELISA.

**FIGURE 3:**
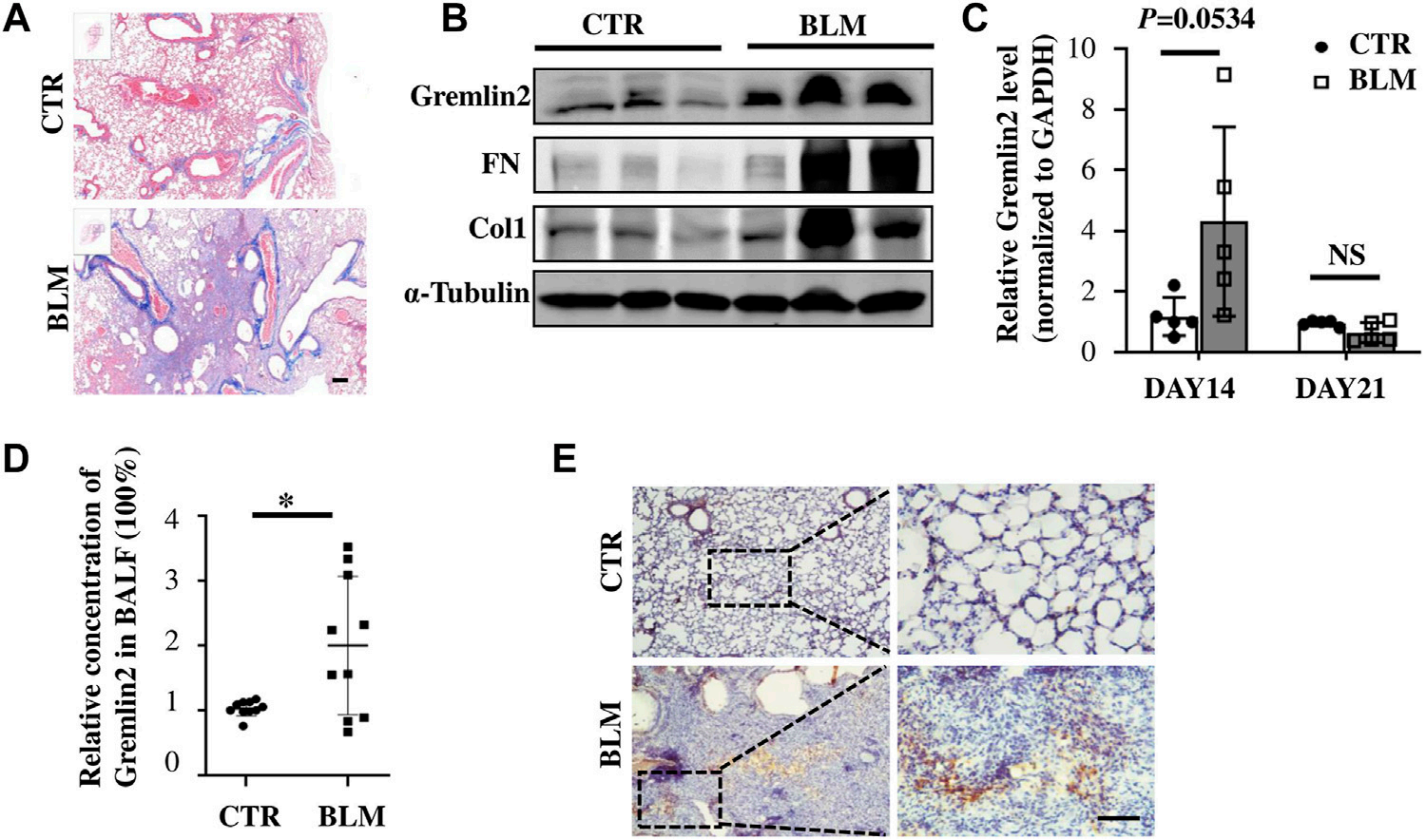
Increased expression of Gremlin2 in the lung tissue of bleomycin-induced pulmonary fibrosis mice **(A)** Masson’s trichrome staining of the lung tissue in the bleomycin-treated mice and PBS-treated control mice. **(B)** 21 days after bleomycin (BLM) or PBS (CTR) treatment mice were sacrificed. Western blotting was performed using the lung tissue homogenates. α-tubulin was used as a reference collagen1 (Col1) and fibronectin (FN) were detected as indicators of fibrosis, and Gremlin2 (Grem2) was used as the target protein. **(C)** Following the same operation method as **(B)**, the corresponding mRNA was extracted from the lung tissue of mice treated for 14 and 21 days, and real-time-quantitative PCR was used to detect the mRNA expression levels of Gremlin2. Day 14 CTR *n* = 5, BLM *n* = 5, *p* = 0.0534, student’s paired t-test; day 21 CTR *n* = 5, BLM *n* = 5, NS *p* > 0.05, student’s paired t-test. **(D)** Expression level of Gremlin2 in alveolar lavage fluid. CTR *n* = 10, BLM *n* = 10, *p = 0.0102, student t paired test. **(E)** Representative images of control lung tissue sections (CTR) and fibrotic lung tissue (BLM) assessed using immunohistochemical staining with anti-Gremlin2 antibody. The right two pictures are 10 x magnified versions of the left two corresponding pictures. The relative content of Gremlin2 was obtained after standardized numerical treatment. Use the Student’s bilateral t-test. Scale bar = 50 μm. CTR, control with PBS-treated group; and BLM, bleomycin-treated group.

Bleomycin airway infusion (2.5 U/kg) was used to induce pulmonary fibrosis in mice, we detected fibrosis induction using Masson trichromatic staining (Figure 3A). The blue area represented collagen accumulation, suggesting a fibrotic condition. The expression of Gremlin2 in the lung tissue was detected using western blotting (Figure 3B). Compared with the commonly used fibrosis indexes involving collagen1 (Col1) and fibronectin (FN), we found that the Gremlin2 expression level in the fibrosis model mice group increased more significantly, and was even more sensitive and indicative than the international indexes.

By detecting the mRNA level of Gremlin2 in the lung tissues at different time points, we found that the transcription level of Gremlin2 increased significantly on day 14, while there was no statistical difference between the pulmonary tissues of fibrotic mice and the control group on day 21 (Figure 3C, *p* = 0.0534). This suggested that the transcription level of Gremlin2 increased during the acute progression of fibrosis, thus affecting the process. Gremlin2 content of mouse alveolar lavage fluid was traced using ELISA. As shown in Figure 3D, the concentration of Gremlin2 in the alveolar lavage fluid was significantly increased (*p* = 0.0102). Using IHC, we observed that the expression of Gremlin2 in the fibrotic lung tissue was significantly increased (Figure 3E). The time course (day 0, day 7, day 14, day 21) data of Gremlin2 mRNA, protein, and BALF were shown in Supplementary Figure S2. Overall, Gremlin2 expression was high in the bleomycin-induced pulmonary fibrosis mouse model.

### Myofibroblasts Express High Levels of Gremlin2 in Bleomycin-Induced Lung Fibrosis Mouse Model

**FIGURE 4:**
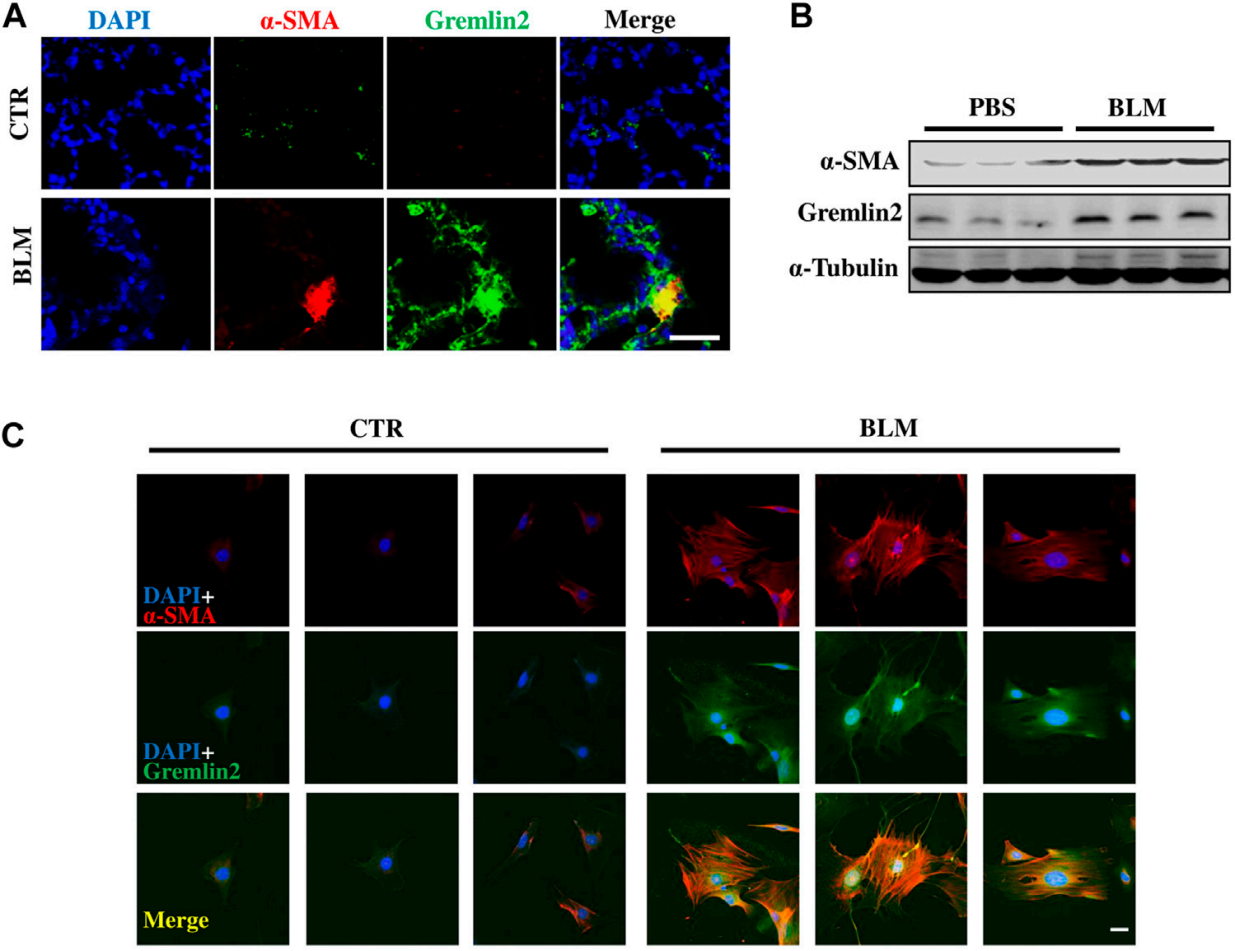
Myofibroblast expresses high level of Gremlin2 in bleomycin induced lung fibrosis model through co-localization **(A)** Immunofluorescence shows co-localization of fibroblast activation marker (α-SMA) and Gremlin2 in lungs of bleomycin-induced fibrosis mice, Gremlin2 (Green), and α-SMA (Red), DAPI (Blue), Yellow (Merge), scale bar = 10 μm. **(B)** Fibroblasts were isolated from mice after PBS or bleomycin treatment on day 14, then western blotting was performed to detect the expression of Gremlin2, and α-SMA. Every group contained three mice. CTR *n* = 3, BLM *n* = 4. **(C)** Following the same operation method as **(B)**, then immunofluorescence was performed to detect the expression of Gremlin2 (Green), and α-SMA (Red). DAPI (Blue) indicated the nucleus. Scale bar = 20 μm. CTR *n* = 3, BLM *n* = 4. CTR, control with PBS-treated group; and BLM, bleomycin-treated group.

To investigate whether the cells with high Gremlin2 expression were activated fibroblasts or not, immunofluorescence staining was used. It was difficult to identify the precise location of Gremlin2, as it is secreted. Co-localization of α-SMA and Gremlin2 in bleomycin-induced fibrosis lung of mice was observed (Figure 4A).

Then we isolated lung fibroblasts on day 14 after treatment with PBS or bleomycin to detect Gremlin2 expression. After culturing for 5 days, we examined the expression of α-SMA and Gremlin2 in lung fibroblasts through western blotting (Figure 4B) and immunofluorescence detection (Figure 4C). Figures 4B,C both show the high relevance between the expressions of α-SMA and Gremlin2, indicating myofibroblasts were the effector cells of Gremlin2 expression.

### Gremlin2 is an Activated Regulator to Fibroblasts

**FIGURE 5:**
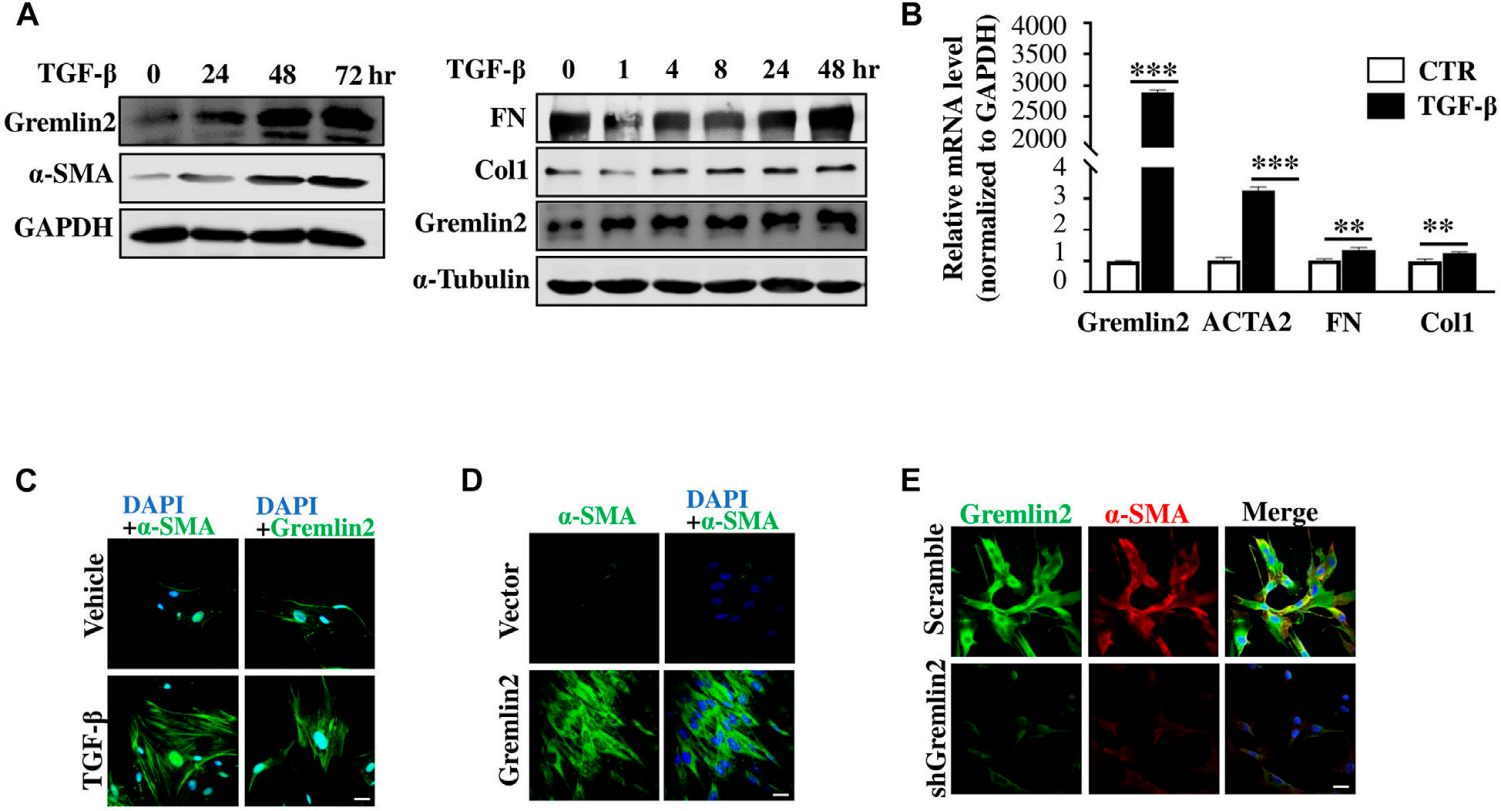
Gremlin2 is an activated regulator to fibroblast **(A)** HFL-1 were collected after treatment with TGF-β1 at 5 ng/mL for 0, 24, 48, and 72 h. Protein expression was detected using western blotting, with GAPDH as a quantitative indicator protein, α-SMA as an indicator of fibroblast activation, and Gremlin2 as a target protein (this experiment was repeated three times). The same treatment applied to HFL-1 cells with TGF-β1 at 5 ng/mL for 0, 1, 4, 8, 24, and 48 h. Western blotting was used to detect fibronectin and collagen1 expression. α-Tubulin was used as the quantitative indicator protein. **(B)** The mRNA was extracted after treatment of HFL-1 cells with TGF-β1 (5 ng/mL) for 24 h, and then real-time quantitative PCR was performed to detect the corresponding mRNA expression levels. α-SMA (ACTA2), fibronectin (FN), and collagen1 (Col1) were used as indicators of fibroblast activation (this experiment was repeated three times). Gremlin2 ***p < 0.0001, Acta2 ***p = 0.0002, FN **p = 0.0037, Col1 **p = 0.0069. **(C)** Immunofluorescence staining was performed on HFL-1 cells were treated with 5 ng/mL TGF-β1 for 24 h to reveal the content of target antigens in the cells. Blue (DAPI) represents the nucleus, green represents the target antigen (α-SMA/Gremlin2). The control group (CTR) received the same treatment without TGF-β1 (the same experiment was performed using HFL-1). Scale bar = 20 μm. **(D)** Gremlin2 was overexpressed in HFL-1, the control group was transfected with empty-load pcDNA3.1, and TGF-β1 stimulation was performed. Immunofluorescence staining was used to detect the content of target antigens in the cells, with DAPI representing the nucleus and α-SMA representing the target antigen in green as an indicator of fibroblast activation. Scale bar = 20 μm. **(E)** Gremlin2 knockdown in MRC-5 cells using shGremlin2, the control group transfected with plko.1. Immunofluorescence staining showed DAPI in blue indicating the nucleus, Gremlin2 in green, and α-SMA in red. Scale bar = 20 μm. All the experiments have been repeated three times.

We used TGF-β1 (Li et al., 2014; Meng et al., 2016) to stimulate fibroblasts and mimic the activation of fibroblasts *in vivo* (Xie et al., 2018). Protein and mRNA levels of Gremlin2 were measured after TGF-β1 treatment in fibroblasts (Figures 5A,B). Gremlin2 expression increased significantly upon activation of fibroblasts, and the mRNA levels increased in line with the ECM production (*p* < 0.05). The well-known markers of fibroblast activation, including FN, Col1, and α-SMA (ACTA2) were chosen to verify the activation. Similarly, α-SMA and Gremlin2 staining was used to detect expression levels with or without TGF-β1 treatment (Figure 5C). We obtained similar results using immunofluorescence and western blotting, where TGF-β1 activated fibroblasts showed upregulated Gremlin2 expression.

Next, we wanted to explore whether Gremlin2 expression affected the activation of fibroblasts. First, α-SMA expression was detected in fibroblast via cellular immunofluorescence (Figure 5D). We found that after Gremlin2 overexpression, fibroblasts were spontaneously activated into myofibroblasts. The expression of α-SMA in human fetal lung fibroblast 1 (HFL-1) (ATCC Cat# CCL-153, RRID:CVCL_0298) cells or MRC-5 (ATCC Cat# CCL-171, RRID:CVCL_0440) showed high fluorescence intensity after Gremlin2 overexpression. However, fluorescence intensity did not increase further in Gremlin2-overexpressing HFL-1 cells after TGF-β1 stimulation, which might indicate that Gremlin2 and TGF-β1 counteract each other in fibroblast activation, as shown in Supplementary Figure S3.

To explore the effect of Gremlin2 deficiency, we used an adenovirus containing shGremlin2 and detected changes in fibroblasts stimulated by TGF-β1 after Gremlin2 knockdown (Figure 5E). After knockdown of Gremlin2, α-SMA expression decreased simultaneously, and fibroblasts were significantly less activated by TGF-β1 than control fibroblasts. Overall, these might indicate that the presence of Gremlin2 was crucial for fibroblast activation.

We obtained the same results from primary lung fibroblasts of bleomycin-induced lung fibrosis mouse model and activated HFL-1 cells. We observed the same upward trend in α-SMA and Gremlin2 expressions. Compared to classic lung biopsy for IPF diagnosis, it was more convenient to detect Gremlin2 expression from BALF, which could be used as a detection method to indicate the occurrence and development of fibrosis.

### Gremlin2 Regulates Migration and Invasion of Fibroblasts

**FIGURE 6:**
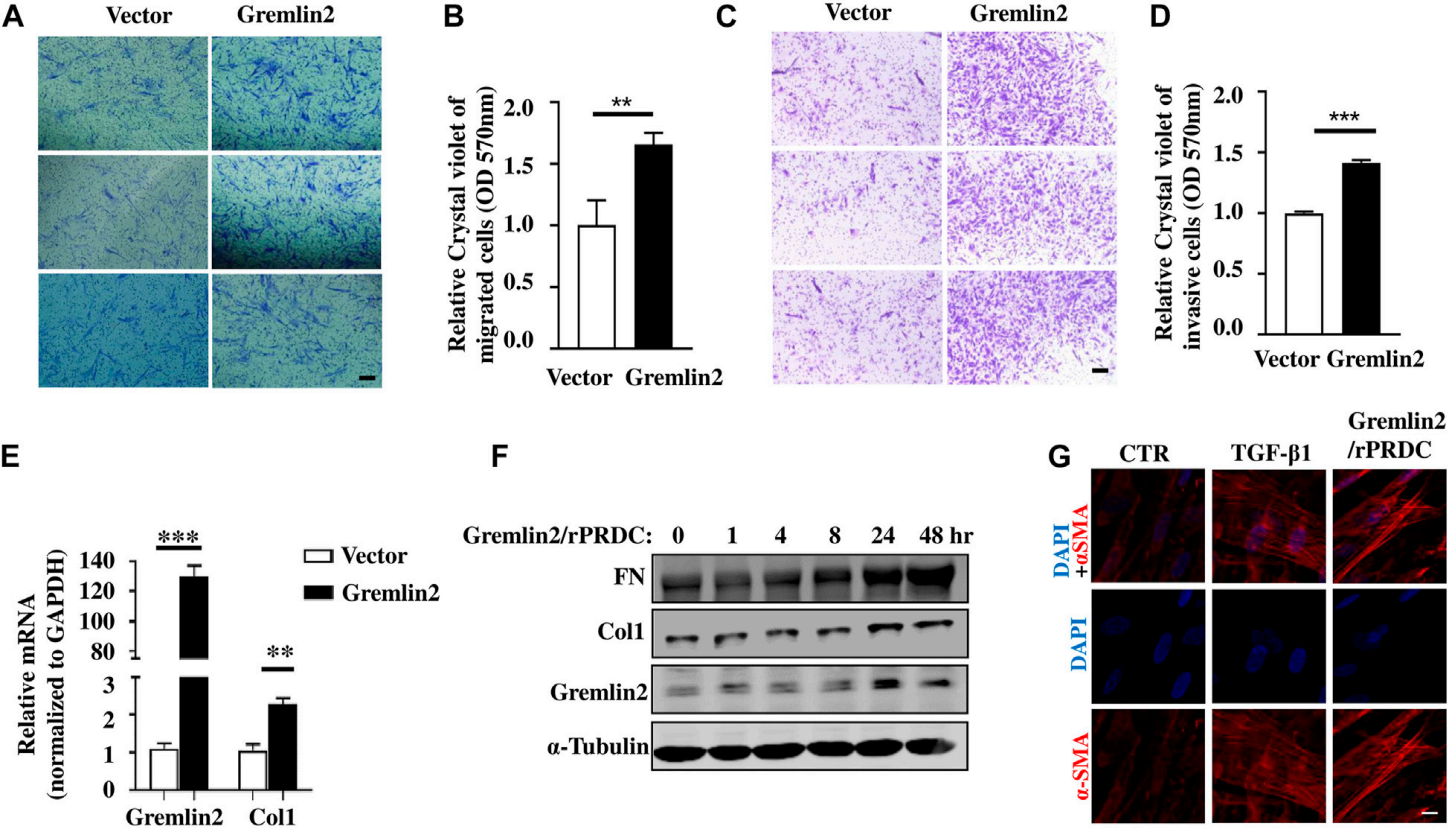
Overexpression of Gremlin2 increases the migration and invasion of fibroblasts. **(A)** Transwell cell migration experiment after Gremlin2 overexpression in fibroblasts. The lower chamber was stained with crystal violet 24 h later, and three fields were randomly selected for imaging under 100× magnification. Scale bar = 100 µm. The experiment was repeated three times. **(B)** The value of OD570 after elution of crystal violet, normalized to that of the control group, **p = 0.072. Unpaired student t test. **(C)** Transwell cell invasion experiment after 1:32 matrix glue was laid. After 48 h, crystal violet staining was carried out on the lower layer of the chamber. Scale bar = 100 µm. The experiment was repeated three times. **(D)** The value of OD570 after elution of crystal violet, normalized to that of the control group. ***p < 0.001. Unpaired student t test. **(E)** HFL-1 cells transfected with Gremlin2 expression plasmid for 24 h, and mRNA was extracted. Then, real-time quantitative PCR was performed to detect the corresponding mRNA expression levels. Using student's t-test, ***p < 0.001, **p < 0.01. Scale bar = 100 µm. **(F)** Recombinant human PRDC (rPRDC)/GREM2 was added to HFL-1 cells for 0, 1, 4, 8, 24, and 48 h, then western blotting was performed to detect the expression of fibronectin, collagen1, and Gremlin2. **(G)** TGF-β1 or recombinant human PRDC (rPRDC)/GREM2 was added to HFL-1 cells for 48 h, then immunofluorescence was performed to detect the expression of α-SMA (Red). DAPI (Blue) shows the nucleus. Scale bar = 20µm. All the experiments have been repeated three times.

Next, we identified the functional role of Gremlin2 in lung fibroblasts by investigating whether Gremlin2 expression regulates the invasion and migration of fibroblasts. Gremlin2 overexpression in lung fibroblasts showed a significant increase in fibroblast migration (Figures 6A,B; *p* = 0.0024) and invasion (Figures 6C,D; *p* = 0.0054). We also examined Col1 mRNA expression levels and found that there was a strong correlation between Gremlin2 and Col1 expression (Figure 6E). This suggested that Gremlin2 might control migration and invasion by increasing ECM production. To identify the function of Gremlin2, we used the recombinant human Gremlin2/rPRDC to stimulate HFL-1 cells. Gremlin2/rPRDC activated fibroblasts, inducing high expression of FN and Col1 (Figure 6F). α-SMA expression also increased, which was similar to TGF-β1 stimulation (Figure 6G). These results revealed that high expression of Gremlin2 in the activated fibroblasts affected their migration and invasion, and Gremlin2 acted on the inactive fibroblasts after secretion, exerting a positive feedback effect.

### Gremlin2 Activates Fibroblasts Through the Bone Morphogenic Protein Signaling Pathway

**FIGURE 7:**
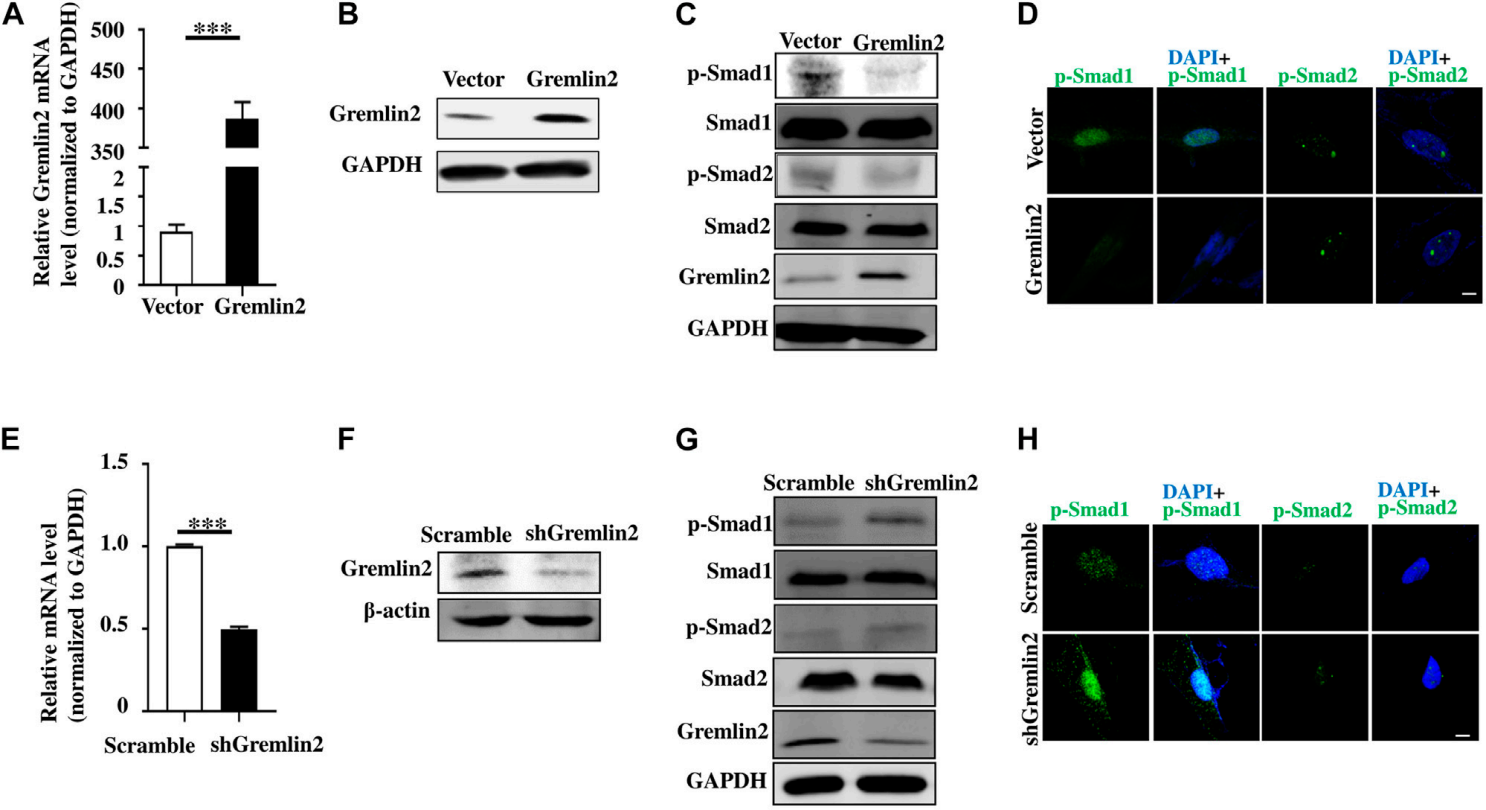
Gremlin2 regulates fibroblast activation through the TGF-β/BMP signaling pathway. **(A)** The mRNA level of Gremlin2 after HFL-1 cells were transfected with Gremlin2 or empty-load plasmid for 24 h. **(B)** The expression level of Gremlin2 after HFL-1 were transfected with Gremlin2 or empty-load plasmid for 72 h detected using western blotting. **(C)** Detection of the p-Smad1 and p-Smad2 in the control group and fibroblasts overexpressing Gremlin2, Smad1 and Smad2 were used as the overall control group. **(D)** Expression level of p-Smad1 and p-Smad2 in the control group and fibroblasts overexpressing Gremlin2 using immunofluorescence. Green represents p-Smad1 and p-Smad2, and blue represents DAPI (nucleus). Scale bar = 5µm. **(E)** The mRNA expression of Gremlin2 after HFL-1 cells were treated with shRNA of Gremlin2 for 24 h, and the control group was infected with plko.1 no-load virus without the target gene, and the corresponding mRNA expression level was standardized to that of GAPDH. **(F)** Protein concentration in the cells was detected using western blotting with actin as the quantitative indicator protein and Gremlin2 as the target protein. **(G)** Expression level of p-Smad1 and p-Smad2 in the control group and the Gremlin2 knockdown HFL-1 cells with Smad1 and Smad2 as the overall control. **(H)** The expression of p-Smad1 and p-Smad2 in the control group and Gremlin2 knockdown fibroblasts, and the expression level of target antigen. Green represents p-Smad1 and p-Smad2 and blue represents DAPI (nucleus). Scale bar = 5 m. ***p < 0.001, Student's t-test bilateral was used to analyze. Experiments were repeated three times.

We used Gremlin2 overexpression plasmid in fibroblasts and verified it at mRNA and protein levels (Figures 7A,B; *p* < 0.001). When Gremlin2 was overexpressed in fibroblasts, phosphorylation of Smad1 was decreased, while phosphorylation of Smad2 was not significantly changed (Figure 7C). To visualize the changes in Smad1 and Smad2 phosphorylation, we detected *p*-Smad1 and *p*-Smad2 through immunofluorescence. The fluorescence intensity in the nucleus represented the level of phosphorylation. The results showed that Gremlin2 overexpression affected the Smad1 phosphorylation, leading to low expression of *p*-Smad1. However, Gremlin2 overexpression had an insignificant effect on Smad2 phosphorylation (Figure 7D).

To explore the effect of Gremlin2 deficiency, we infected fibroblasts with Gremlin2-specific adenovirus and validated the knockdown efficiency at transcription and translation levels (Figures 7E,F). When Gremlin2 was knocked down, Smad1 phosphorylation increased, while Smad2 phosphorylation did not significantly change. To visualize the change in Smad1 and Smad2 phosphorylation, we located *p*-Smad1 and *p*-Smad2 through immunofluorescence. The results showed that Gremlin2 knockdown upregulated the phosphorylation of Smad1, but had little effect on the phosphorylation level of Smad2 (Figure 7H). As shown in Figures 7E−H, the HFL-1 fibroblast cell line was selected for Gremlin2 knockdown, and fibroblast activation was not performed during this process. Even in the inactive state, Gremlin2 knockdown also affected the Smad1 phosphorylation level, indicating that baseline Gremlin2 expression might regulate the BMP signaling. To prove that Gremlin2 affects Smad1 phosphorylation, we knocked down Gremlin2 in TGF-β1-activated HFL-1 cells, and the same trend was observed (Figure 7G). Smad1 phosphorylation was upregulated in activated fibroblasts (Supplementary Figure S4).

Overall, overexpression of Gremlin2 in HFL-1 cells affected the phosphorylation of Smad1 to mediate the BMP signaling pathway.

## Discussion

Here, we achieved overexpression of Gremlin2 (gain of function) and downregulation of Gremlin2 using specific shRNA (loss of function), which showed that Gremlin2 knockdown upregulated the phosphorylation of Smad1 and Gremlin2 overexpression downregulated the phosphorylation of Smad1. In the mice model, the phosphorylation of Smad1 in primary lung fibroblast was upregulated in the bleomycin treatment group shown in Supplementary Figures S6E−G. These results suggest that Gremlin2 might regulate fibrosis via controlling the phosphorylation of Smad1 to mediate the BMP signaling pathway.

IPF is a progressive, fatal disorder that presents a major challenge for clinicians, and the mechanisms that control IPF are not fully understood (King et al., 2011). The main pathological manifestations of IPF are characteristic fibroblast foci composed of major effector fibroblasts (ATS/ERS, 2000). There have been many studies on fibroblasts, including the senescence phenotype of fibroblasts (Waters et al., 2018), tumor-related fibroblasts (Woodcock et al., 2019), and activation of fibroblasts. Myofibroblasts are key effector cells involved in ECM deposition in various fibrotic conditions, including IPF. Our studies demonstrated the profibrotic role of Gremlin2 in pulmonary fibrosis. We provided data at the cellular, molecular, and animal levels to support a role of Gremlin2 as a therapeutic target candidate for lung fibrosis.

First, through the known databases GSE10667 and GSE99621, we found that the expression of Gremlin2 in the fibrotic lung tissue was significantly higher than that in the healthy tissue. Second, Gremlin2 mRNA and protein levels were high in IPF lung tissues and blood serum compared to healthy people. The data collected from the GEO database, including GSE10667 and GSE99621, are shown in Figure 1, while all the samples collected from patients primarily diagnosed with IPF or those who had a lung transplant due to interstitial pulmonary disease with fibrosis are shown in Figure 2. The GSE10667, the dataset contained data of samples collected after a biopsy or lung transplant from patients with IPF at the University of Pittsburgh Health Sciences Tissue Bank. The GSE99621 dataset contained the samples of healthy lung tissues from healthy controls and affected or unaffected lung tissues from patients with IPF. Because of the low incidence of IPF (2–60 cases per 100,000 persons per year) (Martinez et al., 2017), it was difficult to collect enough samples of lung tissue samples to meet the IPF diagnosis standard. As the focus of our research was Gremlin2 expression in myofibroblasts and fibroblasts around fibrotic lesions, which are pathological features of the fibrotic region, we collected the fibrotic lesion transplant samples not only from patients with IPF but also from those with other types of lung fibrosis. Therefore, we defined the collective tissue samples as pulmonary fibrosis (PF) samples. Third, when fibroblasts were treated with TGF-β1, Gremlin2 expression increased, which correlated with ECM production. When the expression of Gremlin2 was inhibited through shRNA knockdown, ECM production decreased. We verified the function of upregulating Gremlin2 in mice lung fibroblast in Supplementary Figure S6. The primary lung fibroblasts in bleomycin treatment mice lung showed the high capacity of migration and invasion shown in Supplementary Figures S6A−D. One hypothesis has emerged that links IPF and cancer due to their similar hallmark pathological alterations, such as aberrant myofibroblast proliferation and apoptosis and ECM invasion (Vancheri et al., 2010). Studies showed that cell invasion increases in IPF fibroblasts (Li et al., 2011). Moreover, IPF lung fibroblasts have an increased migratory capacity compared to healthy lung fibroblasts (Cai et al., 2010). We showed here that Gremlin2 overexpression in lung fibroblasts increased the migration and invasion of them. Although we showed that Gremlin2 was upregulated in IPF lung tissues and myofibroblasts, but the role of Gremlin2 in alveolar type II cells was not investigated.

Co-labeling via immunofluorescence showed that Gremlin2 was expressed not only in myofibroblasts but also in the injured epithelial cells. Based on the results of Figure 4A, these α-SMA-negative cells expressing gremlin2 might have involved epithelial cells, shown in Supplementary Figure S1. We used E-Cadherin as an indicator of epithelial cells and co-localized it with Gremlin2 immunofluorescence staining in mice fibrosis lung tissue. Supplementary Figure S1 showed epithelial cells could express Gremlin2. We selected different visual fields for analysis. Supplementary Figure S1A showed some epithelial cells expressed Gremlin2 with the white arrow pointing to the merge part. Supplementary Figure S1B was hardly any cell co-expressed Gremlin2 and E-Cadherin, indicating that there were other types of cells expressing Gremlin2 in the fibrotic area and not all epithelial can express Gremlin2. Overall, the epithelial cells, possibly the injured epithelial expressed Gremlin2, indicating that injured epithelial cells might play role in the process of fibrosis. This suggested that Gremlin2 might cause the process of pulmonary fibrosis through the migration and invasion of fibroblasts and epithelial injury repair. In this article, we focused on the function of Gremlin2 in fibroblasts; meanwhile, the role of Gremlin2 in epithelial injury repair should also be investigated.

Type II alveolar epithelial (AT-II) cells play a key role in the regulation of alveolar physiology (Zhang et al., 2012). Dysfunctional AEC2 should regenerate damaged cells, leading to insufficient repair ability. Persistent epithelial injury is a key mechanism of severe fibrosis in patients with IPF (Liang et al., 2016). Therefore, exploring the function of Gremlin2 in alveolar epithelial cells might reveal if Gremlin2 is involved in other aspects of fibrosis as well.

Here, we provided another hypothesis that injured epithelial cells expressing Gremlin2 might be another possible mechanism of IPF.

The TGF-β superfamily, which includes the BMP family, is an important regulator of fibrosis (Henderson et al., 2020). In bleomycin-induced lung fibrosis, TGF-β and BMP signaling follow an inverse course, with dynamic activation of TGF-β signaling and repression of BMP signaling. Modulating the balance between BMP and TGF-β may be a therapeutic target in fibrotic lung disease (De Langhe et al., 2015). BMP-7 expression decreases in patients with IPF (Gu et al., 2014) and BMP-7 supplementation significantly reduces the hydroxyproline content in mice treated with aseptic materials (Myllärniemi et al., 2008a). Strong antagonists of the BMP family also include Gremlin1 and CHORD. Previous studies have shown that Pirfenidone can reduce the activity of fibroblasts induced by TGF-β, and the upregulation of BMP4/Gremlin1 can be detected (Jin et al., 2019), while Gremlin-1 regulates the recruitment of inflammatory cells and the production of anti-fibrosis chemokines in the lung (Koli et al., 2016).

However, we also recognized there are a few limitations to the current investigation. Studies showed that Fstl1 promoted TGF-β signaling and inhibits BMP signaling in epithelial cells, whereas in fibroblasts Fstl1 promotes TGF-β signaling without altering BMP signaling (Geng et al., 2011; Zheng et al., 2017). Therefore, further investigation into the mechanisms under which Gremlin2 regulates lung fibrosis could provide a novel target for the development of therapeutics for patients with pulmonary fibrosis. Gremlin2 is a secreted glycoprotein, meaning that it is not only expressed in a specific area but also can be detected in the bloodstream. As a development-related protein, it is highly expressed during embryonic development but its expression is low in adulthood. The high expression of it in adults might imply a severe disease state. We detected the concentration of Gremlin2 in the blood serum of healthy control samples, and the low expression verified that Gremlin2 low-expression was stable in the healthy control group. The increase in the expression of Gremlin2 can be easily detected via its increased secretion into the bloodstream. Moreover, compared to the golden criteria of a lung biopsy for IPF diagnosis, the detection of biomarkers in the blood serum is more convenient for patients. Less invasive techniques are more appliable in IPF screening at the early stage, therefore, biomarkers, such as Gremlin2, should be further investigated for their potential clinical application.

Besides, in mice model, we found that Gremlin2 showed an upregulated tendency after 2.5 units of bleomycin treatment with time going on. More interestingly, the concentration of Gremlin2 in BALF after 5 units of bleomycin treatment showed an exaggerated increase on day 7 shown in Supplementary Figure S5A. We tried to explore the origin by immunohistochemistry and cell immunofluorescence. By immunofluorescence co-staining CD11b and Gremlin2, we found a significant co-staining relationship between them showed in Supplementary Figure S5B in the 5 units bleomycin-treated 7 days group. But the control group treated with PBS showed no this phenomenon shown in Supplementary Figure S5C. Furthermore, we got the cells in BALF from the bleomycin-treated mouse group, the Giemsa staining showed a great amount of macrophage shown in Supplementary Figure S5D. Cell immunofluorescence co-staining CD11b and Gremlin2 also showed the great consistency shown in Supplementary Figure S5E. Besides, we constructed the one-day model treated by LPS for the acute inflammation model to explore the neutrophils would express Gremlin2 or not. The result showed in Supplementary Figure S5F may indicate the low expression of Gremlin2 in neutrophils. In all, we found the macrophage may contribute to the high expression of Gremlin2 in BALF of the 5-units bleomycin-treated group. Mice treated with 5 units of bleomycin began to die after 7 days showed high expression of Gremlin in BALF. Gremlin2 was first found as a development-related protein, the high expression of Gremlin2 in adulthood might indicate a severe disease condition. Our experiments have confirmed that fibroblasts can be activated by Gremlin2 to form myofibroblasts shown in Figures 6F,G, and a large amount of Gremlin2 production in early macrophages may contribute to the activation of fibroblasts and the progression of fibrosis.

We have constructed Gremlin2 knockout mice and will continue to explore the role of Gremlin2 in pulmonary fibrosis in future studies. Further, we have confirmed that Gremlin2 caused the increased invasive fibroblasts phenotypes, but more *in vivo* evidence is needed to verify that it affects mouse lung fibrosis through myofibroblasts. α-SMA-positive myofibroblasts or Col1a1-positive fibroblasts both originate from cells expressing Tbx4, can be applied as a general marker of fibroblasts (Xie et al., 2016). TBX4-creER, Gremlin2^flox/flox^ mouse model would further verify the role of Gremlin2 in promoting fibrosis through mesenchymal cells.

## Data Availability

The datasets presented in this study can be found in online repositories. The names of the repository/repositories and accession number(s) can be found in the article/Supplementary Material.

## Supplementary Material

Supplementary Figure S1 Epithelial cells express Gremlin2. Immunofluorescence shows the co-localization of the epithelial cell marker E-Cadherin and Gremlin2 in the lungs of bleomycin-induced fibrosis mice. Gremlin2 (Green), and E-Cadherin (Red), DAPI (Blue), Yellow (Merge), scale bar = 20 μm. **(A)** The white arrow pointed to the co-labeled cells. **(B)** The high expression of Gremlin2 or E-Cadherin.

Supplementary Figure S2 Gremlin2 expression at mRNA and protein levels and its levels in BALF of mice on day 0, 7, 14, 21 after bleomycin treatment. The relative concentration of Gremlin2 in the BALF, and its expression at mRNA and protein levels in the lung tissues of mice on **(A)** day 0 (CTR, *n* = 5; BLM, *n* = 5), **(B)** day 7 (CTR, *n* = 5; BLM, *n* = 4), **(C)** day 14 (CTR, *n* = 5; BLM, *n* = 5), **(D)** day 21 (BALF CTR, *n* = 10; BLM, *n* = 10, mRNA and protein levels CTR *n* = 5, BLM *n* = 5) after bleomycin (BLM) or PBS (CTR) treatment. Student’s unpaired t-test.

Supplementary Figure S3 Gremlin2 is overexpressed in HFL-1 cells. The control group was transfected with empty-load pcDNA3.1 with or without TGF-β1 treatment. Immunofluorescence staining was used to detect the content of target antigens in the cells, with DAPI representing the nucleus and α-SMA representing the target antigen in green as an indicator of fibroblast activation. Scale bar = 20 μm.

Supplementary Figure S4 Gremlin2 activates fibroblasts through the BMP signaling pathway. Expression level of p-Smad1 and p-Smad2 in the control group and the Gremlin2-knockdown HFL-1 cells after 48 h of TGF-β1 treatment with Smad1 and Smad2, as the overall control.

Supplementary Figure S5 Macrophages contribute to the high expression of Gremlin2 in BALF. **(A)** The relative concentration of Gremlin2 in BALF at day7 days after 5 units bleomycin (BLM) or PBS (CTR) treatment mice were sacrificed. CTR *n* = 5, BLM *n* = 4, student’s unpaired t-test. *p* = 0.0073. **(B)** Gremlin2 (Green) and CD11b (Red) were co-stained by immunofluorescence of bleomycin **(a)** or PBS **(b)** treated mouse lungs. The right pictures are the enlargement of the white frame area in the left picture. White scale bar = 100 μm, yellow scale bar = 20 μm. **(C)** The Giemsa staining of cells from mice BALF. Black scale bar= 20μm. **(D)** Gremlin2 (Green) and CD11b (Red) were co-stained by immunofluorescence of cells in BALF from bleomycin treated mice. The right pictures are the enlargement of the white frame area in the left picture. White scale bar = 40 μm, yellow scale bar = 10 μm. **(E)** Gremlin2 (Green) and CD11b (Red) were co-stained by immunofluorescence of cells in BALF from one-day LPS treated mice. The right pictures are the enlargement of the white frame area in the left picture. White scale bar = 40 μm, yellow scale bar = 10 μm.

Supplementary Figure S6
*p*-Smad1 is upregulated in activated mice lung fibroblasts after bleomycin treatment. **(A)** Transwell cell migration experiment of mice lung fibroblasts after PBS or bleomycin treatment. The lower chamber was stained with crystal violet 24 h later, and three fields were randomly selected for imaging under 100x magnification. Scale bar =100 μm. CTR *n* = 2, BLM *n* = 2. **(B)** The value of OD570 after elution of crystal violet, normalized to that of the control group, ***p* = 0.001. Unpaired student t test. **(C)** Transwell cell invasion experiment of mice lung fibroblasts after PBS or bleomycin treatment. The lower chamber was stained with crystal violet 24 h later, and three fields were randomly selected for imaging under 100x magnification. Scale bar = 100 μm. CTR *n* = 2, BLM *n* = 2. **(D)** The value of OD570 after elution of crystal violet, normalized to that of the control group, ****p* < 0.001. Unpaired student t test. **(E)** Detection of the p-Smad1 and p-Smad2 in the fibroblasts from PBS or bleomycin treatment group, Smad1 and Smad2 were used as the overall control group. CTR *n* = 3, BLM *n* = 3. Paired student t test was used, *p* = 0.0011. **(F)** Expression level of p-Smad1 in the fibroblasts from PBS or bleomycin treatment group using immunofluorescence. Green represents p-Smad1 and p-Smad2, and blue represents DAPI (nucleus). Scale bar = 10 μm. CTR *n* = 3, BLM *n* = 5. Scale bar = 10 μm. **(G)** Expression level of p-Smad2 in the fibroblasts from PBS or bleomycin treatment group using immunofluorescence. Green represents p-Smad1 and p-Smad2, and blue represents DAPI (nucleus). Scale bar = 10 μm. CTR *n* = 3, BLM *n* = 5. Scale bar = 10 μm. CTR presented as PBS treatment group, BLM presented as the bleomycin treatment group.
