# Supplementary figures and images for: Gremlin2 Activates Fibroblasts to Promote Pulmonary Fibrosis Through the Bone Morphogenic Protein Pathway
Source: Front Mol Biosci. 2021 Jun 28;8:683267. doi: 10.3389/fmolb.2021.683267 (PMC8377751; doi:10.3389/fmolb.2021.683267)

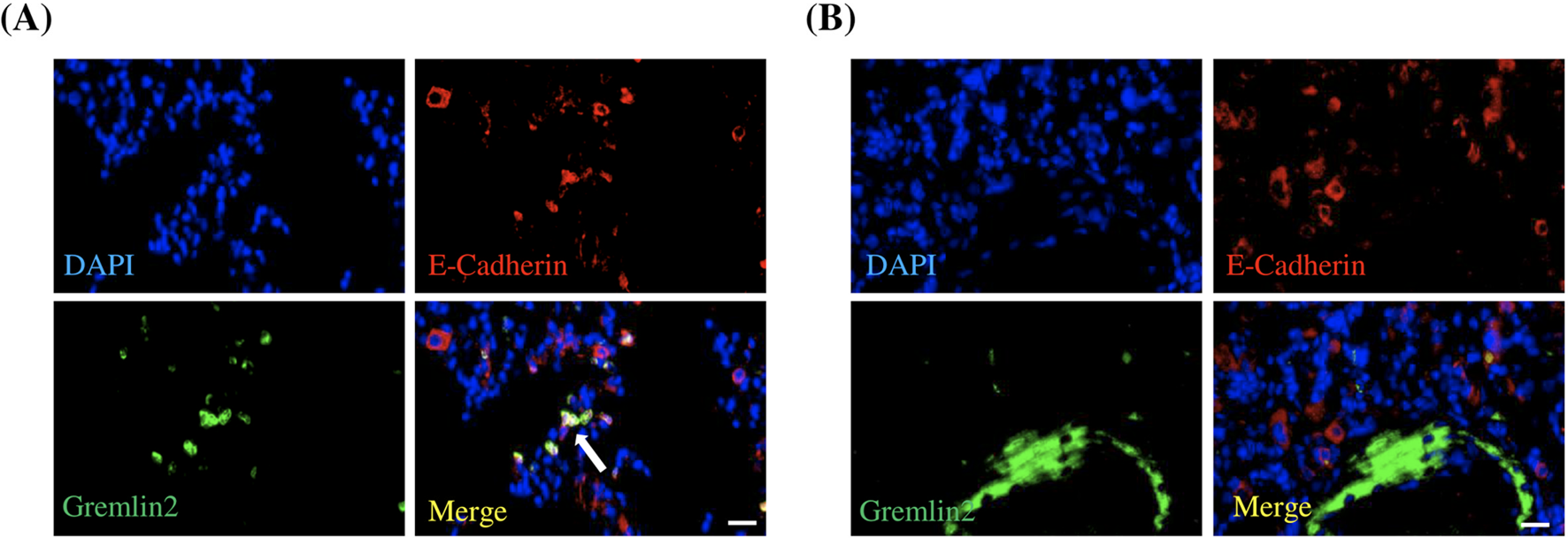

Supplement: Supplementary file 1 [file Image1.tif]

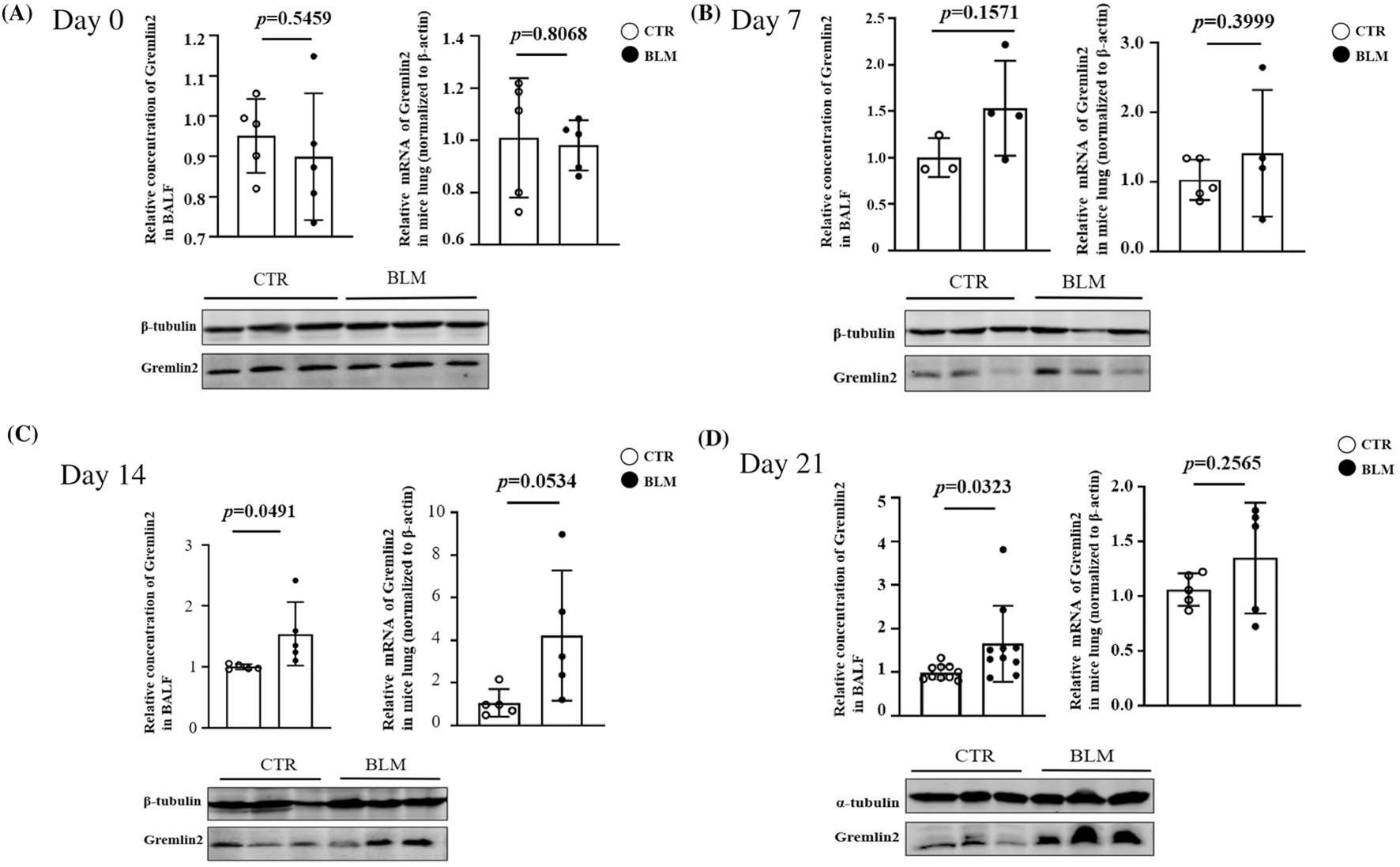

Supplement: Supplementary file 2 [file Image2.tif]

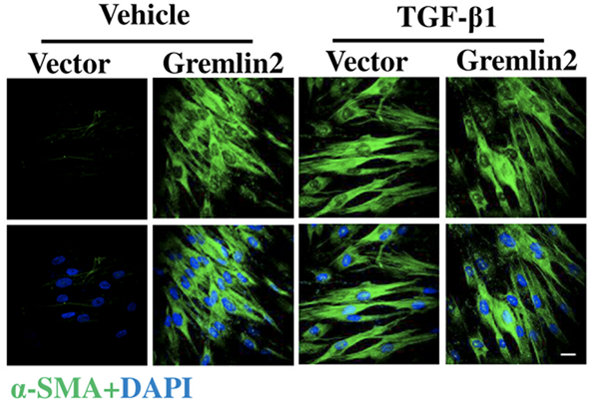

Supplement: Supplementary file 3 [file Image3.tif]

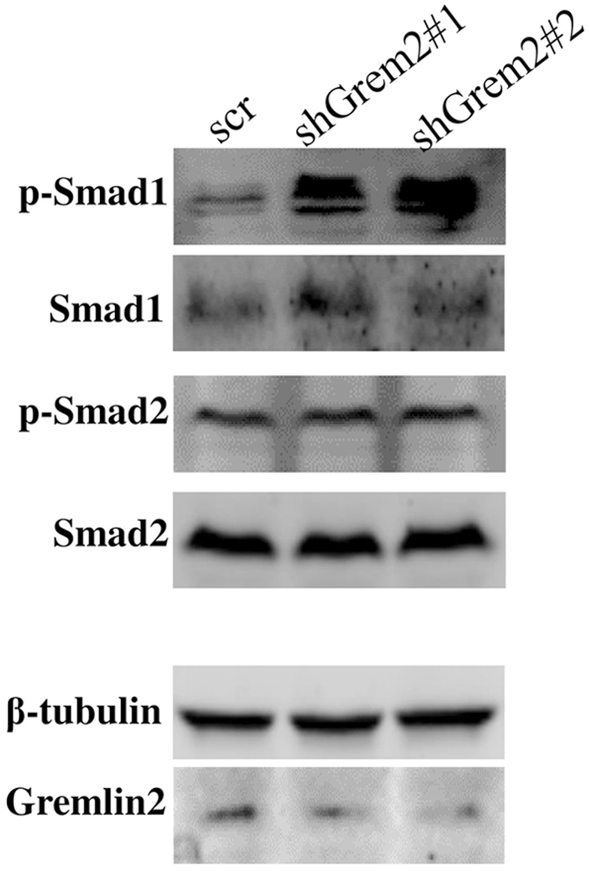

Supplement: Supplementary file 4 [file Image4.tif]

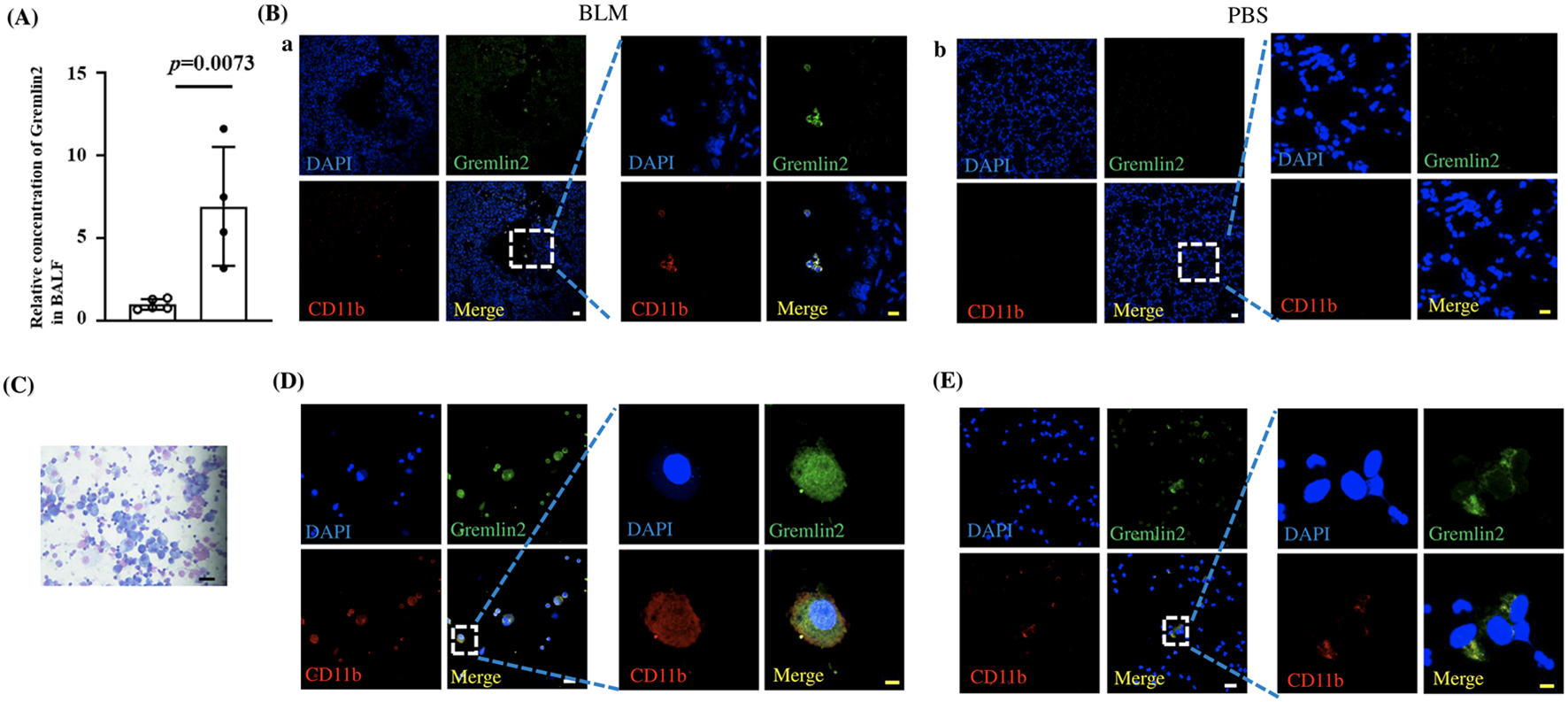

Supplement: Supplementary file 5 [file Image5.tif]

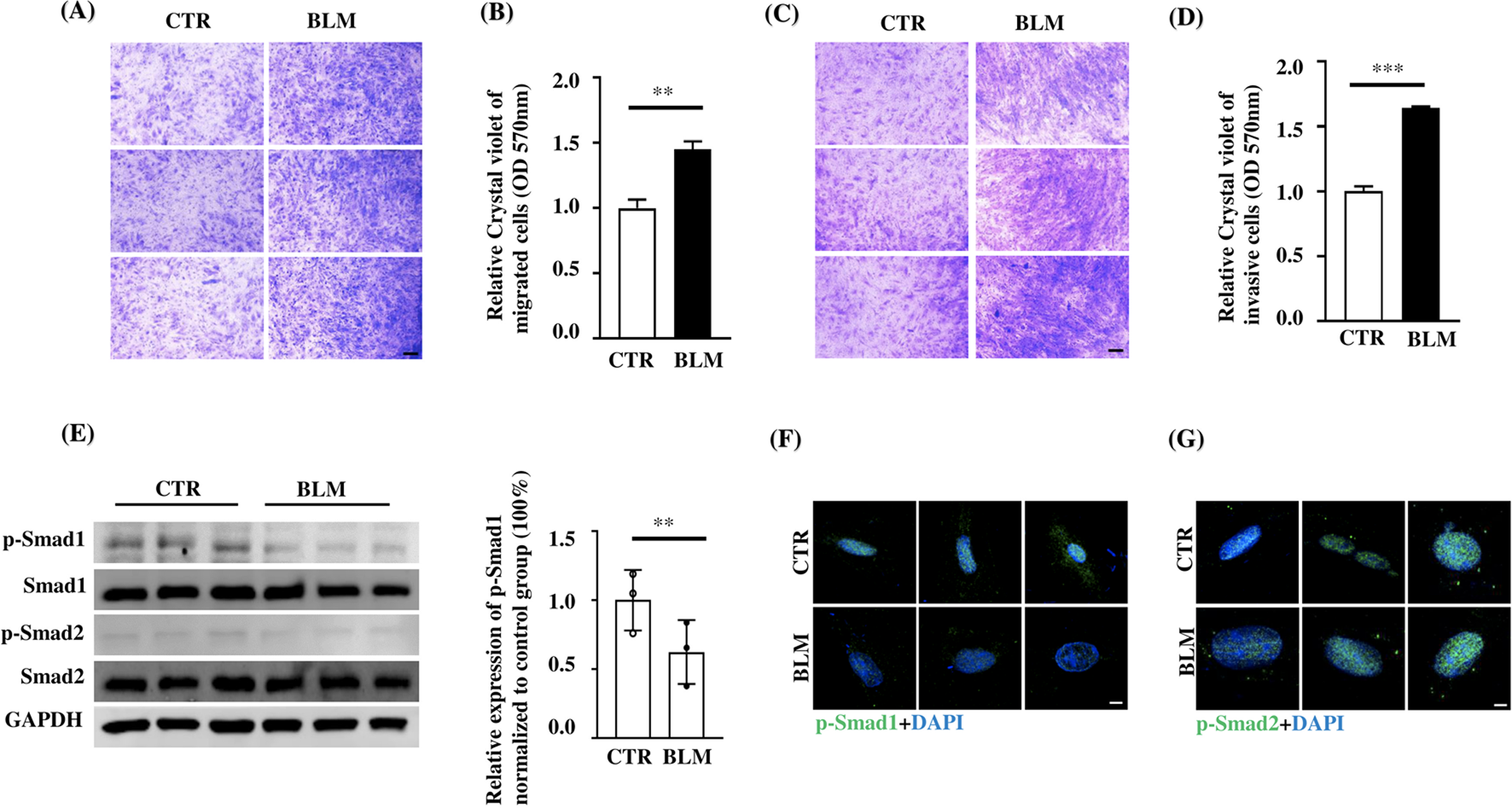

Supplement: Supplementary file 6 [file Image6.tif]
